# Utility of contrast-enhanced magnetic resonance lymphangiography for yellow nail syndrome with lymphangiopathy: a case report

**DOI:** 10.1093/omcr/omac077

**Published:** 2022-07-26

**Authors:** Yui Shimanuki, Shion Miyoshi, Nanami Anzai, Yusuke Usui, Nobuyuki Shiraga, Kazuma Kishi

**Affiliations:** Department of Respiratory Medicine, Toho University Omori Medical Center, Ota-ku, Tokyo, Japan; Department of Respiratory Medicine, Toho University Omori Medical Center, Ota-ku, Tokyo, Japan; Department of Respiratory Medicine, Toho University Omori Medical Center, Ota-ku, Tokyo, Japan; Department of Respiratory Medicine, Toho University Omori Medical Center, Ota-ku, Tokyo, Japan; Department of Radiology, Toho University Omori Medical Center, Ota-ku, Tokyo, Japan; Department of Respiratory Medicine, Toho University Omori Medical Center, Ota-ku, Tokyo, Japan

## Abstract

Yellow nail syndrome (YNS) is a rare disorder characterized by the triad of yellow nails, lymphedema and chronic respiratory manifestations. Lymphatic abnormalities are a characteristic finding of YNS. Nevertheless, proof of lymphatic vessel abnormality by direct needle puncture for contrast agent injection is technically challenging because the lymphatic vessels in YNS are dysplastic. Thus, we opted for contrast-enhanced magnetic resonance (MR) lymphangiography with subcutaneous injection in patients suspected of YNS to facilitate easier comprehensive lymphatic vessel visualization. The lymphatic vessels of the thighs were few and barely recognizable, indicating weak flow cranially and lymphatic vessel hypoplasia. These findings were suggestive of dysplasia of the lymphatic vessels. Therefore, MR lymphangiography may be a useful novel diagnostic modality for YNS.

## INTRODUCTION

Yellow nail syndrome (YNS) is a rare disorder characterized by the triad of yellow nails, lymphedema and chronic respiratory manifestations, which include pleural effusion [1]. Its diagnosis is based on clinical manifestations and exclusion of malignancy, infection and inflammatory disease. Although the pathophysiology of YNS is unknown, structural and functional lymphatic system abnormalities have been implicated [2]. The lymphatic vessels in YNS are dysplastic; so, comprehensive imaging by direct needle puncture for contrast agent injection is technically challenging. Thus, we opted for contrast-enhanced magnetic resonance (MR) lymphangiography with subcutaneous injection, which facilitates easier comprehensive lymphatic vessel visualization.

**Figure 1:**
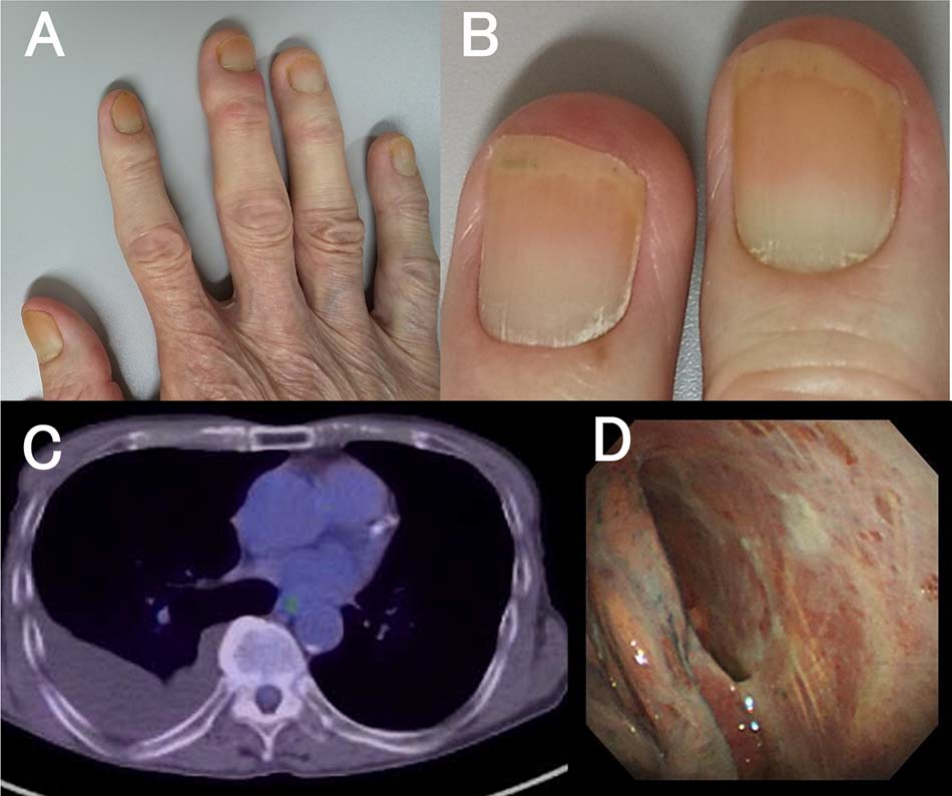
Delayed growth with yellow nail discoloration (**A**, **B**). 18-Fluorodeoxyglucose positron emission tomography showing no significant pleural uptake (**C**). Pleuroscopic findings showing no tumor and non-specific inflammatory changes (**D**).

## CASE REPORT

A 70-year-old male was admitted to our hospital because of a cough that persisted for several months and fluid accumulation in the right pleural cavity. He had no previous medical history of malignant disease and supplement or medication use. All nails showed yellow discoloration with delayed growth (Fig. 1A and B). He had mild bilateral pitting pedal edema and negative Stemmer sign; unremarkable blood tests; and normal tumor marker levels and interferon gamma release assays.

High-resolution chest-computed tomography showed right pleural effusion and bronchiectasis. In addition, 18-Fluorodeoxyglucose positron emission tomography revealed no uptake within the pleura (Fig. 1C).

He underwent pleuroscopy under local anesthesia to evaluate the pleural lesions and rule out malignant disease. A patchy white pleural thickness was revealed using pleuroscopic findings (Fig. 1D). The pleural effusion appeared yellow and was exudative effusion with lymphocytic predominance. Pleural fluid cultures were negative results, including for acid-fast bacteria. Pathological findings on pleural biopsy showed non-specific inflammation compatible with fibrotic pleurisy. These results raised suspicion for YNS.

**Figure 2:**
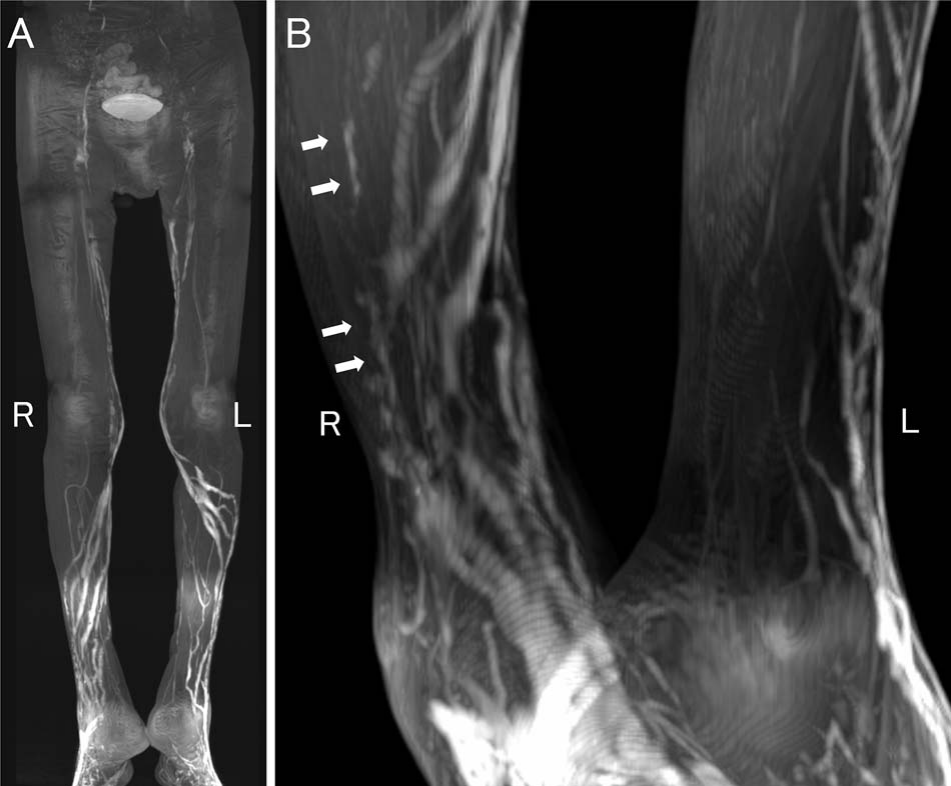
Contrast-enhanced MR lymphangiography 35 min after contrast medium injection showing irregular lymphatic vessels in the lower limbs. (**A**) Full image of the lower limbs, (**B**) Magnified image at the lower leg-level, R-right, and the L-left. Barely identifiable lymphatic vessels in the thighs, indicating weak flow cranially and vessel hypoplasia (A). Few irregular tortuous longitudinal lymphatic vessels varying in caliber in both legs (arrows) (**B**).

Lymphatic vessel irregularities have been reported in YNS, and so we first attempted ultrasound-guided intranodal lymphangiography via direct puncture of the inguinal lymph nodes. However, puncturing was technically difficult because of atypical lymph node atrophy. Next, we attempted puncturing the lymphatic vessels from the dorsum of the foot via a skin incision but the vessels were too small. Alternatively, we performed contrast-enhanced MR lymphangiography with subcutaneous injection of 1 ml of a mixture of gadolinium-based contrast medium (Gadoteridol) and 1% lidocaine (Gadoteridol and 1% lidocaine are mixed in a ratio of 9:1) into the dorsum of the foot. We performed four dynamic phase acquisitions at 5, 15, 25 and 35 min after subcutaneous injection. The lymphatic vessels of the thighs were few and barely identifiable, indicating poor flow cranially and lymphatic vessel hypoplasia at 35 min after subcutaneous injection (Fig. 2A). There were few irregular tortuous longitudinal lymphatic vessels of varying caliber in the region of both legs (Fig. 2B). These findings were suggestive of dysplasia of the lymphatic vessels and lymph nodes; thus a diagnosis of YNS was made.

The patient was treated for YNS with 200 mg per day of clarithromycin and oral vitamin E preparation, but with no therapeutic effect. Eventually, he rapidly became emaciated and developed recurrent aspiration pneumonia. The patient died of respiratory failure 6 months after the diagnosis of YNS.

## DISCUSSION

YNS is associated with structural and functional lymphatic system abnormalities [2]. However, most reports of these lymphatic abnormalities are based on pathological findings at autopsy or indirect findings on lymphatic scintigraphy [3, 4]. To our knowledge, this is the first report on contrast-enhanced MR lymphangiography demonstrating high-accuracy comprehensive imaging of lymphatic vessel abnormalities in a patient with YNS.

Proof of lymphatic vessel abnormality has been based on a diagnostic technique involving direct puncture of the inguinal lymph nodes or lymphatic vessels of the dorsum of the foot and contrast medium injection [5, 6]. However, the lymphatic vessels and nodes in YNS are typically hypoplastic, so this puncture technique requires high-level skills. In addition, there has been a previous report of non-enhanced MR lymphangiography in YNS patients [7], still, it has problems with field heterogeneity and radiofrequency labeling inefficiency, making it difficult to visualize normal or hypoplastic lymphatic vessels [8]. Therefore, we conducted a contrast-enhanced MR lymphangiography by subcutaneous injection as an alternative, easily implementable imaging modality [9].

The examination of contrast-enhanced MR lymphangiography was simple, needing only a subcutaneous injection of MR imaging (MRI) contrast medium into the dorsum of the foot, followed by an MRI scan. The major advantage of contrast-enhanced MR lymphangiography compared with non-enhanced MR lymphangiography is the ability to clearly delineate abnormal lymphatic vessels due to the high spatial and temporal resolution. The pre-contrast sequence was performed in our patient, but lymphatic vessels could not be delineated with a low signal on T1-weighted images. Moreover, MR lymphangiography is preferable to lymphatic scintigraphy as it better depicts lymphatic vessel morphology that gives a more comprehensive picture of vessel abnormalities [10]. Regarding side effects, our patient experienced only mild local pain and swelling due to the subcutaneous injection. There were no serious adverse events such as skin necrosis.

Thus, our patient’s lymphatic vessels had various abnormalities, including dilatation and stenosis. There were fewer lymphatic vessels than in healthy subjects, suggesting lymphatic hypoplasia. Only mild edema of the lower extremities was revealed in the physical examination, although lymphangiography exhibited abnormal manifestations. Contrast-enhanced MR lymphangiography can be useful in lymphedema cases that are challenging to diagnose based on clinical findings alone, as in our patient.

Definitive diagnosis and treatment of YNS are often challenging, and it is likely underdiagnosed. Lymphatic abnormalities are considered a characteristic finding, and MR lymphangiography may be a useful novel diagnostic modality for YNS. However, further accumulation of cases is needed.

## CONFLICT OF INTERESTS

The authors declare that they have no conflict of interest.

## FUNDING

None declared.

## ETHICAL APPROVAL

No ethical approval was needed for this case report.

## CONSENT FOR PUBLICATION

We obtained written informed consent from the wife of the patient after he passed away.

## GUARANTOR

None declared.
